# Deletion at 2q14.3 is associated with worse response to TNF-α blockers in patients with rheumatoid arthritis

**DOI:** 10.1186/s13075-019-1983-y

**Published:** 2019-08-28

**Authors:** Ki-Nam Gu, So-Young Bang, Hye-Soon Lee, Youngho Park, Ju-Yeon Kang, Ji-Soong Kim, Bora Nam, Hyun-Seung Yoo, Jung-Min Shin, Yeon-Kyung Lee, Tae-Han Lee, Sehwan Chun, Soo-Kyung Cho, Chan-Bum Choi, Yoon-Kyoung Sung, Tae-Hwan Kim, Jae-Bum Jun, Dae Hyun Yoo, Kwangwoo Kim, Sang-Cheol Bae

**Affiliations:** 1 Department of Biology, Kyung Hee University, Seoul, 02447 South Korea; 2 Department of Rheumatology, Hanyang University Hospital for Rheumatic Diseases, Seoul, 04763 South Korea; 3 Department of Business Statistics, Hannam University, Daejeon, 34430 South Korea

**Keywords:** Rheumatoid arthritis, Copy number variations, TNF-α blockers, Drug efficacy

## Abstract

**Background:**

Structural variations such as copy number variations (CNVs) have a functional impact on various human traits. This study profiled genome-wide CNVs in Korean patients with rheumatoid arthritis (RA) to investigate the efficacy of treatment with TNF-α blockers.

**Methods:**

A total of 357 Korean patients with RA were examined for the efficacy of TNF-α blocker treatment. Disease activity indexes were measured at baseline and 6 months after the treatment. The patients were classified as responders and non-responders based on the change in disease activity indexes according to the EULAR response criteria. CNVs in the same patients were profiled using fluorescence signal intensity data generated by a genome-wide SNP array. The association of CNVs with response to TNF-α blockers was analyzed by multivariate logistic regression accounting for genetic background and clinical factors including body mass index, gender, baseline disease activity, TNF-α blocker used, and methotrexate treatment.

**Results:**

The study subjects varied in their responses to TNF-α blockers and had 286 common CNVs in autosomes. We identified that the 3.8-kb deletion at 2q14.3 in 5% of the subjects was associated with response to TNF-α blockers (1.37 × 10^− 5^ ≤ *P* ≤ 4.07 × 10^− 4^) at a false discovery rate threshold of 5%. The deletion in the identified CNV was significantly more frequent in the non-responders than in the responders, indicating worse response to TNF-α blockers in the deletion carriers. The 3.8-kb deletion at 2q14.3 is located in an intergenic region with the binding sites of two transcription factors, MAFF and MAFK.

**Conclusions:**

This study obtained the CNV landscape of Korean patients with RA and identified the common regional deletion associated with poor response to treatment with TNF-α blockers.

**Electronic supplementary material:**

The online version of this article (10.1186/s13075-019-1983-y) contains supplementary material, which is available to authorized users.

## Introduction

Rheumatoid arthritis (RA) is a chronic autoimmune disease that primarily affects multiple joints, causing tenderness, heat, swelling, and joint deformity. As persistent inflammation in RA leads to severe joint damage and disability, it is important for RA patients to receive early treatment to improve the symptoms and to achieve remission of disease.

Combination treatments of multiple drugs such as disease-modifying antirheumatic drugs (DMARDs; e.g., methotrexate) and biologics (e.g., TNF-α blockers) have widely been used to treat RA. The TNF-α blockers, a recombinant antibody to TNF-α or its receptor including adalimumab, etanercept, golimumab, and infliximab have proven highly successful in potently suppressing both inflammation and joint disability [1–3]. However, a degree of the response to the TNF-α blockers varies from patient to patient. About 30–40% of patients treated with TNF-α blockers do not effectively respond to the therapy [1, 2, 4], missing other potentially effective treatments at an early stage of disease.

There have been several studies to identify genetic markers to explain the efficacy of biologics treatment in RA. Candidate-gene approaches focused on the genetic effects of known RA-risk loci including *HLA-DRB1* [5] and the members of TNF signaling pathway including *TNF* [6, 7] but failed to identify strong associations with the response of TNF-α blockers. Similarly, a few of genome-wide association studies (GWAS) suggested several SNPs associated with disease activity score (DAS)-based response to biologics treatment but their genetic significance levels did not surpass the genome-wide significance threshold [7–10]. Although all the previous GWAS revealed that genetic effects on response to TNF-α blockers were modest, the GWAS-suggesting variants were highlighted at relevant biological pathways including TNF-α signaling and inflammation pathways [7–10].

In this present study, we further investigated genetic contribution to drug efficacy of TNF-α blockers by analyzing copy number variation (CNV) in the RA patients treated with TNF-α blockers. CNV is the most common structural variation defined as large (> 1 kb) genomic deletions and duplications and could yield a high impact on various traits including drug response by altering the dosage of functional genes and regulatory elements [11–13]. In contrast to single nucleotide polymorphisms (SNPs) in GWAS, CNVs have not yet been investigated for their effects on the response of TNF-α blockers in patients with RA. Here, we newly identified a novel CNV that explained a proportion of the inter-individual variance in efficacy of biologics based on the common response criteria.

## Methods

### Subjects and drug response estimation

A total of 357 Korean RA patients treated by TNF-α blockers were recruited from Hanyang University Hospital for Rheumatic Diseases (Seoul, South Korea). All the study subjects were examined for both drug efficacy of TNF-α blockers and genome-wide CNVs. Clinical and descriptive characteristics in the study subjects are listed in Additional file 1: Table S1. Adalimumab, etanercept, golimumab, and infliximab were treated to 60 patients (16.8%), 260 patients (72.8%), 19 patients (5.3%), and 18 patients (5.0%), respectively.

RA disease activity was accessed at baseline and 6 months by the DAS28 that was calculated using 4 variables, including 28 tender-joint count (TJC; range 0–19), 28 swollen-joint count (SJC; range 0–28), erythrocyte sedimentation rate (ESR), and general health (GH) [14]. The EULAR response criteria based on the change in DAS28 (ΔDAS28) after TNF-α blocker therapy [15] were used to classify the degree of response as no improvement, moderate improvement, or good improvement.

Additionally, the disease activity was assessed by the second common disease index called clinical disease activity index (CDAI) for the same subjects. CDAI is a numerical summation (without acute-phase reactant [16]) of the counts of TJC and SJC along with patient and physician global assessment (GA) [17].

Scores from TJC and SJC are relatively large parts of a CDAI value, compared to DAS28 [18]. As both DAS28 and CDAI are the common disease activity indexes, analyses using both the indexes can be useful to check whether the detected CNV response are reliable and consistent without a potential bias from index selection.

### Genetic data for CNV call and quality control

Genome-wide CNVs in the subjects were profiled based on the fluorescence signal intensities from a high-density genome-wide SNP array, Illumina Omni2.5Exome-8 BeadChip microarray containing about 2.5 million probes. To obtain reliable fluorescence signal clusters and CNVs, a CNV analysis was performed in 922 Korean individuals by combining the 370 study subjects and the other 552 out-of-study subjects whose data were generated by the same array in the same experimental batch. We performed a general quality control (QC) for genome-wide SNP data (Additional file 1: Table S2). Briefly, 22 out-of-study individuals were excluded from subsequent analyses due to excessive heterozygosity, excessive singleton, different genetic background, and cryptic relatedness (Additional file 1: Table S2a). For SNPs, we extracted ~ 2.5 million unique SNPs with a call rate per SNP ≥ 95% and a *P* value for Hardy-Weinberg equilibrium (HWE) ≥ 1 × 10^− 6^ in the CNV call (Additional file 1: Table S2b).

### CNV call

The copy number of genomic regions was determined by the PennCNV software applying a hidden Markov model (HMM) algorithm [19]. Genotype clusters of fluorescence signals for each QC-passed variant were generated to calculate individual-level estimates of the total intensity of normalized fluorescence signals in a log scale (log R ratio; LRR) and the fluorescence signal proportion from a minor allele (B allele frequency; BAF), based on population-level cluster [20]. LRRs and BAFs were finally used in calling CNVs, with other several parameters including HMM constants, distances between neighboring SNPs, and the population-level frequency of minor alleles [19]. All detected CNVs were supported by more than three SNPs. Thirteen individuals with the excessive copy number (> 100) were excluded from subsequent analyses.

### Statistical analyses for the associations between CNVs and drug efficacy

**Fig. 1:**
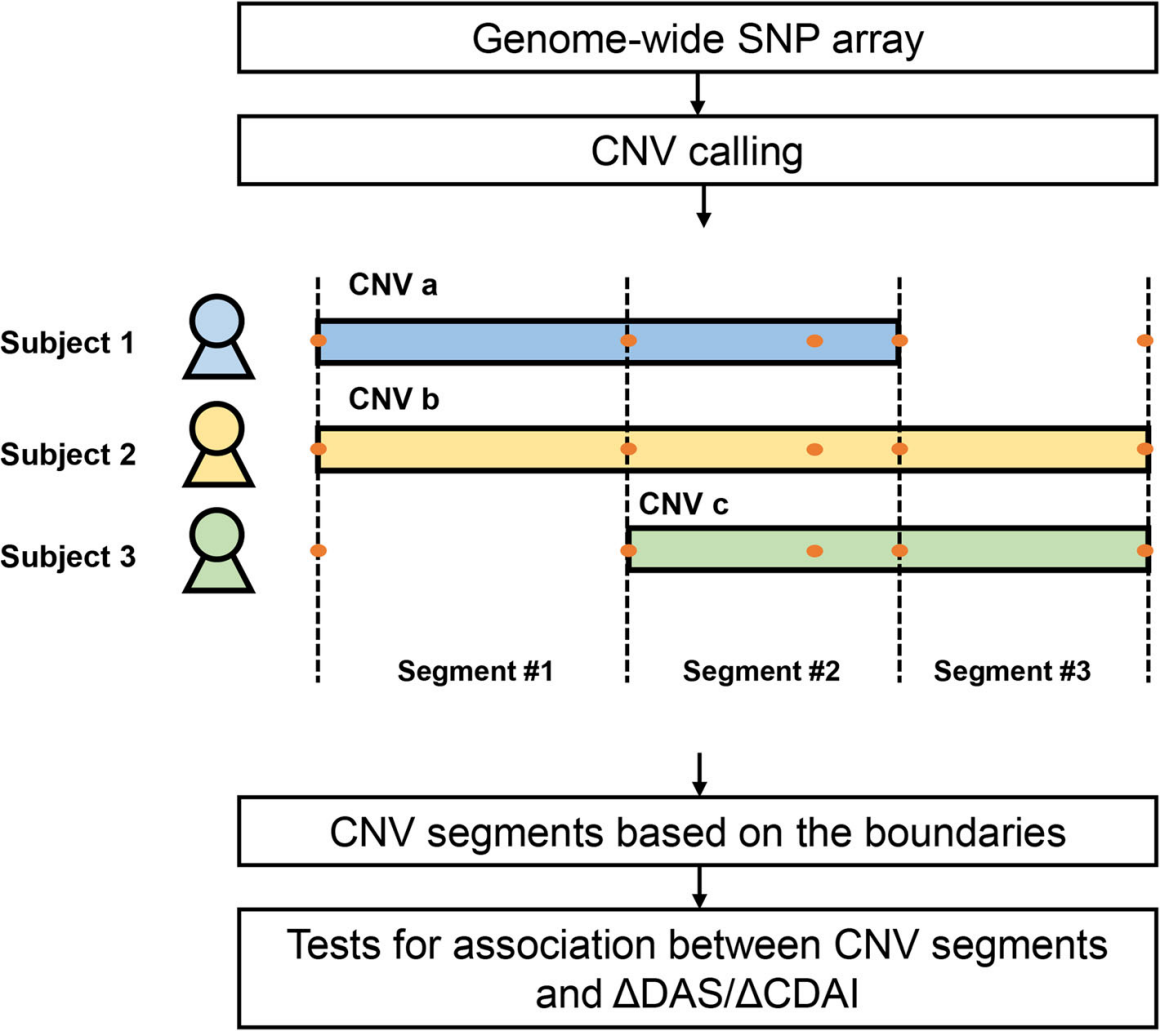
Analysis workflow. Fluorescence signals from a genome-wide SNP array with 2.5 million probes were analyzed to profile CNV in patients with RA. CNVs overlapped across the study subjects were segmented at all CNV boundaries. In the example shown in the figure, CNVs overlapped with different boundaries (determined by fluorescence signals at SNPs) among three subjects were divided into three segments. Different boundaries and SNPs are indicated by dash lines and orange dots, respectively. Associations between CNV segments and response to the treatments were then tested using ΔDAS28 and ΔCDAI

The individual-level CNV profile in the study subjects (*n* = 357) was tabulated, taking into account the different boundaries of CNVs among individuals. CNV regions were split at the CNV boundaries observed in the study subjects and we then counted the copy number of the CNV segments (Fig. 1). The deletion-only and duplication-only CNV segments not following HWE (*P*_HWE_ ≤ 0.05) were excluded, and common CNV segments (*n* = 286) where ≥ 5% of the subjects had abnormal copy numbers were tested for the association with the response criteria defined by ΔDAS28 after 6 months from the baseline of DAS28. Multivariate logistic regression analyses accounting for genetic background and clinical factors including body mass index, gender, baseline DAS28, TNF-α blocker used, and methotrexate treatment were performed to calculate the effect sizes of CNV segments and their standard errors. Significance of associations was determined at a false discovery rate (FDR) threshold of 0.05. Alternatively, we tested for the CNV associations with the change in CDAI (ΔCDAI) by multivariate linear regression with the same covariates (excluding baseline DAS28) and baseline CDAI at an FDR of 5%.

## Results

### Characteristics of study subjects

This study analyzed 357 Korean RA patients treated for TNF-α blockers and examined for CNVs. The descriptive clinical information of the subjects is shown in the “Methods” section and Additional file 1: Table S1. In our study patients, the baseline of DAS28 right before treatment had a mean value of 6.24 with a standard deviation of 0.89. The ΔDAS28 at 6 months after treatment from the baseline DAS28 ranged from − 1.33 to 5.98 in the subjects. We classified the patients into non-responders (*n* = 32; 9%) and responders (208 moderate + 117 good responders; 58% + 33%), according to the EULAR response criteria using the ΔDAS28 [15]. In the same study patients, the baseline of CDAI had a mean value of 32.85 with a standard deviation of 11.83. The ΔCDAI estimated at 6 months after treatment from the baseline CDAI varied from − 22 to 71.5 in the subjects.

### Characteristics of discovered CNVs

We identified 10,604 CNVs in the QC-passed 357 study subjects, divided them into 10,913 CNV segments based on CNV boundaries (Fig. 1), and used 286 common CNV segments with the frequency of abnormal-copy carrier ≥ 5% and *P* for HWE > 0.05 in the subsequent statistical analysis, considering statistical power given the sample size and the lack of HWE at unreliable CNVs. The mean and the median length of the 286 CNV segments were 2.7 kb and 1.4 kb, respectively. We observed that 99 (34.6%) of the common CNVs were characterized by only deletion; 55 (19.2%) were defined by only duplication; and 132 (46.2%) were multi-class CNVs that appeared both deleted and duplicated copy numbers.

### Association of a CNV at 2q14.3 with response to TNF-α blocker therapy

**Fig. 2:**
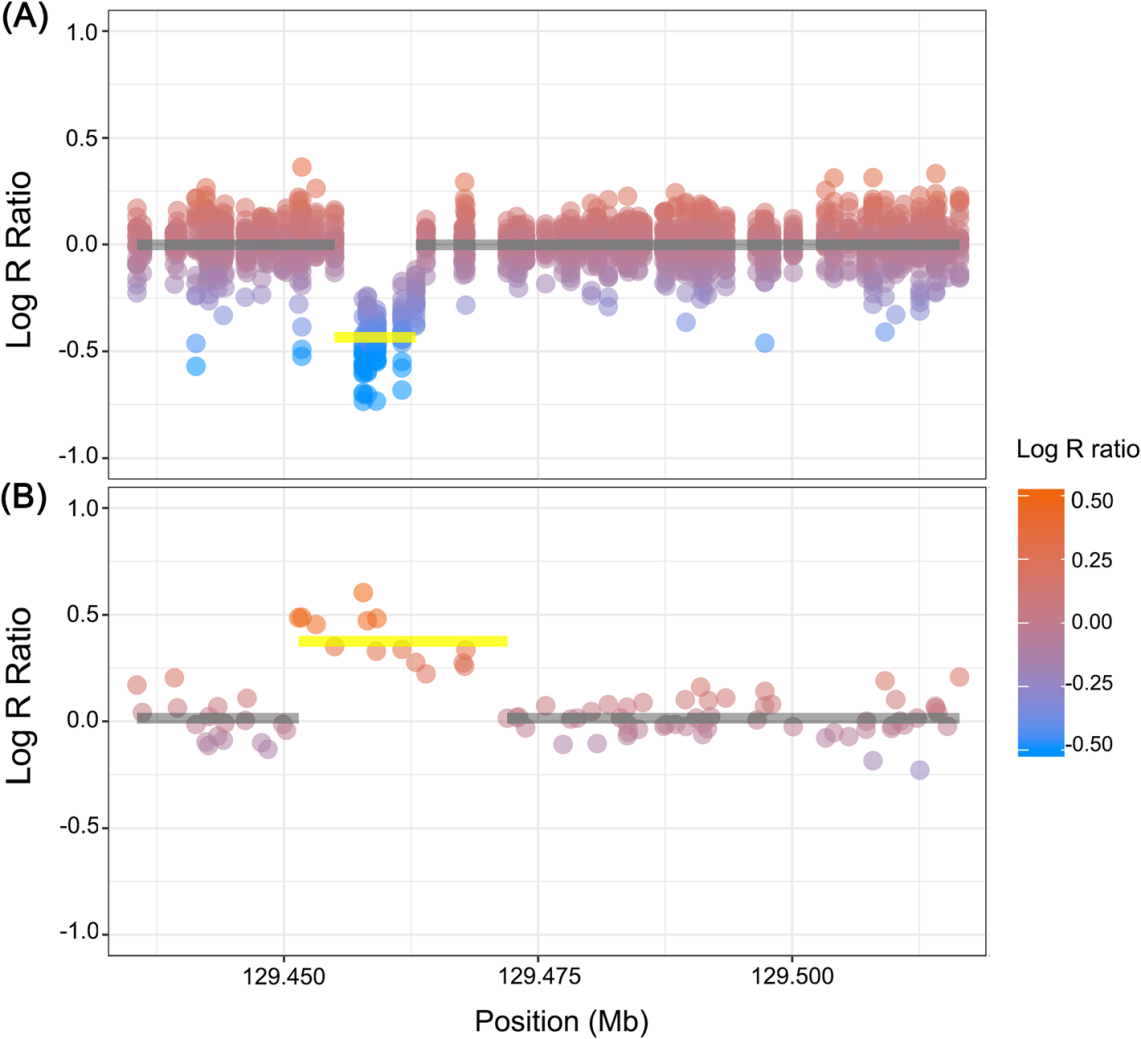
LRR values within and around the CNVs at 2q14.3. **a** LRR in hemizygous-deletion carriers (*n* = 22). **b** One-copy duplication with the increased intensity of fluorescence signals in a duplicated-copy carrier. Yellow lines present the mean of LRR at the deleted and duplicated regions. Gray lines present the mean of LRR at the two-copy regions

We performed a multivariate logistic regression analysis to investigate the association of each of 286 common CNV segments with ΔDAS28-based response to TNF-α blocker therapy, conditioning on the genetic principal components, body mass index, gender, baseline DAS28, TNF-α blocker used, and methotrexate use. We identified the association of three successive CNV segments at 2q14.3 where we detected ≤ 3.8-kb multi-class CNVs with abnormal copy numbers in ≤ 6.2% of the subjects (Fig. 2, Table 1). The CNV region at 2q14.3 was supported by the fluorescence intensity signals of five array SNPs and can be divided into three segments of 2.5-kb, 0.9-kb, and 0.4-kb sizes based on four CNV boundaries in the subjects. The three CNV segments were defined as multi-class CNVs with low-frequency deletion (5.6%, 5.9%, and 6.2%, respectively) and rare duplication (0.2%, 0.2%, and 0.2%, respectively; Table 1). The deletion CNV and five typed SNPs lying within the CNV were under HWE among the patients (*P*_HWE_ ≥ 0.11).

**Table 1:** Association summary statistics of three significant CNV segments at 2q14.3 associated with DAS28-based response to TNF-α blocker therapy in 357 patients with RA

			Non-responders; *n* (%)			Responders; *n* (%)			
Chr	Region	OR of being NR by deletion (95% CI)	CN = 1	CN = 2	CN = 3	CN = 1	CN = 2	CN = 3	*P*
2	129,459,141–129,461,606	8.44 (2.77 to 25.71)	7 (22.9%)	25 (78.1%)	0 (0.0%)	13 (4.0%)	311 (95.7%)	1 (0.3%)	1.67 × 10^−4^
2	129,457,798–129,458,197	7.88 (2.58 to 24.05)	7 (22.9%)	25 (78.1%)	0 (0.0%)	14 (3.8%)	310 (95.4%)	1 (0.3%)	2.72 × 10^−4^
2	129,458,197–129,459,141	7.24 (2.41 to 21.76)	7 (22.9%)	25 (78.1%)	0 (0.0%)	15 (4.2%)	309 (95.1%)	1 (0.3%)	4.07 × 10^−4^

The copy number of 0 or ≥ 4 were not observed in the study subjects

*DAS28* disease activity scores based on 28 joint counts, *Chr* chromosome, *OR* odds ratio, *NR* non-responders, *CI* confidence interval, *CN* copy number

The significance levels for the association of each CNV segment with response surpassed an FDR threshold of 5% (1.67 × 10^− 4^ ≤ *P* ≤ 4.07 × 10^− 4^; Table 1). The loss of copy number in the identified CNV segments was significantly more in the non-responders than in the responders [e.g., for the 2.5-kb CNV segment, odds ratio = 8.44 (95% confidence interval = 2.77 to 25.71)], indicating worse response to TNF-α blockers in the deletion carriers. A duplication observed in an individual spanned the three response-associated CNV segments and five flanking segments in a 16.4-kb region (Fig. 2b).

To further investigate whether the significant genetic association at 2q14.3 is consistent regardless of response indexes, we tested for the CNV association using a widely accepted, alternative disease index, ΔCDAI. The ΔDAS28 and ΔCDAI values in the patients with RA were highly correlated (*r* = 0.83). In multivariate linear regression adjusting for baseline CDAI and the same covariates used in the ΔDAS28 association analysis (except baseline DAS28), we consistently identified the same CNV segments at 2q14.3 associated with ΔCDAI at an FDR threshold of 5%, showing lower *P* values (1.37 × 10^− 5^ ≤ *P* ≤ 7.90 × 10^− 5^; Table 2) than those from ΔDAS28.

**Table 2:** Association summary statistics of three significant CNV segments at 2q14.3 associated with CDAI-based response to TNF-α blocker therapy in 357 patients with RA

			Non-responder; *n* (%)			Responder; *n* (%)			
Chr	Region	Regression coefficient of deletion (95% CI)	CN = 1	CN = 2	CN = 3	CN = 1	CN = 2	CN = 3	*P*
2	129,459,141–129,461,606	8.16 (4.52 to 11.79)	7 (22.9%)	25 (78.1%)	0 (0.0%)	13 (4.0%)	311 (95.7%)	1 (0.3%)	1.37 × 10^−5^
2	129,458,197–129,459,141	7.19 (3.68 to 10.70)	7 (22.9%)	25 (78.1%)	0 (0.0%)	15 (4.2%)	309 (95.1%)	1 (0.3%)	6.97 × 10^−5^
2	129,457,798–129,458,197	7.28 (3.69 to 10.86)	7 (22.9%)	25 (78.1%)	0 (0.0%)	14 (3.8%)	310 (95.4%)	1 (0.3%)	7.90 × 10^−5^

The copy number of 0 or ≥ 4 were not observed in the study subjects

*CDAI* clinical disease activity index, *Chr* chromosome, *CI* confidence interval, *CN* copy number

To check the possibility that the CNV association was observed simply due to the association of flanking SNPs (*n* = 2001), we investigated the association of each SNP 1 Mb around the CNV. None of the flanking SNPs explained the response to TNF-α blockers better than the CNV at 2q14.3. In addition, the maximum correlation *r*^2^ between each SNP and the CNV was very weak (*r*^2^ = 0.045). It strongly indicated that the identified CNV at 2q14.3 was neither tagged nor explained by SNPs, while being independently associated with the drug response to TNF-α blockers.

The phase 3 analysis of the 1000 Genomes Project identified a 6.9-kb common loss CNV including the response-associated 3.8-kb CNV segments at 2q14.3 using a next-generation sequencing technology that can identify the CNV boundaries more accurately than GWAS arrays. Specifically, the frequency of deleted-copy carriers was 5.2% in the East Asian population of the 1000 Genomes Project, not observed in other ethnicities, which is highly consistent with the deletion-carrier frequency in our study subjects (5.6%). Similarly, the maximum correlation *r*^2^ between each of 67,606 flanking SNPs and the CNV was very weak (*r*^2^ = 0.037) in East Asian population. It indicates that the CNV call at 2q14.3 in our study is reliable and the association of the deletion is relevant in response to TNF-α blocker therapy in East Asians.

The CNV segments at 2q14.3 possess no genes but the experimentally validated elements bound by the two transcription factors, MAFF and MAFK, suggesting a *cis*-regulatory effect of the CNV regions on neighboring genes. The nearest gene is *HS6ST1* at the 380-kb upstream of the CNV segments that encodes a member of the heparan sulfate biosynthetic enzyme family. The second nearest gene is *UGGT1* at a 528-kb upstream of the CNV segments encoding the UDP-glucose glycoprotein glucosyltransferase 1.

## Discussion

Treatment with biologics like TNF-α blockers has been common for RA patients, especially the patients with resistance to DMARDs. There are several successful developments of TNF-α blockers including adalimumab, etanercept, golimumab, and infliximab of which efficacy was well-validated based on the changes of disease activity indexes such as ΔDAS28 and ΔCDAI [21–25]. However, the large inter-individual variance in the response to the biologics is poorly understood.

This study conducted the first genome-wide CNV analysis to identify which structural variations including large duplication and deletion were associated with response to the TNF-α blocker therapy. We investigated CNVs in Korean patients with RA treated with TNF-α blockers and their associations with the 6-month response data of ΔDAS28 and ΔCDAI [22, 26]. The association tests revealed that the less copy number at 2q14.3 was associated with poor efficacy of TNF-α blocker therapy in the patients with RA at an FDR threshold of 5% (odds ratio of being a non-responder = 8.44 with a 95% confidence interval = 2.77 to 25.71).

The 3.8-kb CNV segments at 2q14.3 are located at an intergenic region around the 380-kb upstream of *HS6ST1* and the 528-kb downstream of *UGGT1*. As the CNV segments contain the transcription factor binding sites of MAFF and MAFK, it is tempting to suggest that the regions may regulate the gene expression of *HS6ST1* and/or *UGGT1*. Little is known about the biology of these genes in terms of drug response to biologics. *HS6ST1* encodes heparan sulfate 6-O-sulfotransferase 1, of which activity plays a major role in generating a distinct heparan sulfate structure [26]. *UGGT1*, a glycoprotein folding-sensor enzyme, encodes UDP-glucose glycoprotein glucosyltransferase 1 [27] that cooperatively enhances the differentiation of cultured osteoblasts with *FAM5C* [28, 29].

This study was conducted in a single center where all Korean subjects with RA in our analysis were treated with TNF-α blockers and measured for their response in the same clinical practice and protocols. In addition, our analysis utilized both ΔDAS28 and ΔCDAI scores, the most reliable and acceptable indexes to determine disease activity of RA [30]. The response association of the CNV segments at 2q14.3 was most significant in two separate genome-wide association analyses using the ΔDAS28 and ΔCDAI-based response outcomes, indicating little index bias in the association results of the 2q14.3 CNV segments.

There were two major limitations in this study. The statistical power was not sufficient to detect rare CNVs and modest effect sizes due to the small sample size in our analysis. We had to exclude CNVs shown in less than 5% of the samples, considering the statistical power and multiple testing. In addition, as the novel association signal of the identified CNV was not validated in an independent cohort, it needs to be further confirmed in a replication study.

In summary, we identified the large-effect CNV segments that explain the variance of response to the treatment with TNF-α blockers in the Korean patients with RA based on common disease activity indexes—ΔDAS28 and ΔCDAI, suggesting as a potential biomarker to predict efficacy prior to the treatment of TNF-α blockers.

## Additional file

Additional file 1: **Table S1**. Clinical characteristics of the study patients with rheumatoid arthritis. **Table S2**. The number of variants and subjects in a quality control (QC) procedure. (PDF 179 kb)

## Data Availability

The datasets used and/or analyzed during the present study are available from the corresponding authors on request.

## Abbreviations

BAF: B allele frequency
CDAI: Clinical disease activity index
CNV: Copy number variation
DAS: Disease activity score
DAS28: Disease Activity Scores based on 28 joint counts
DMARDs: Disease-modifying antirheumatic drugs
ESR: Erythrocyte sedimentation rate
FDR: False discovery rate
GA: Global assessment
GH: General health
GWAS: Genome-wide association study
HMM: Hidden Markov model
LRR: Log R ratio
QC: Quality control
RA: Rheumatoid arthritis
SJC: Swollen-joint count
SNP: Single nucleotide polymorphism
TJC: Tender-joint count
