# Supplementary material for: Deletion at 2q14.3 is associated with worse response to TNF-α blockers in patients with rheumatoid arthritis
Source: Arthritis Res Ther. 2019 Aug 28;21:195. doi: 10.1186/s13075-019-1983-y (PMC6714408; doi:10.1186/s13075-019-1983-y)
Supplement: Supplementary file 1 — Table S1. Clinical characteristics of the study patients with rheumatoid arthritis. Table S2. The number of variants and subjects in a quality control (QC) procedure. (PDF 179 kb) [file 13075_2019_1983_MOESM1_ESM.pdf]

Table S1 Clinical characteristics of the study patients with rheumatoid arthritis

	All patients	Non-responder	Responder	$P^{\dagger}$
Samples, N	357	32	325	
Age at symptom onset, mean ± SD (years)	41.76 ± 13.62	42.75 ± 14.07	41.66 ± 13.60	0.68
Female, N	312 (87.4%)	27 (84.4%)	285 (87.7%)	0.79
BMI, kg/m^2	22.51 ± 3.53	22.70 ± 4.22	22.50 ± 3.46	0.81
Anti-cyclic citrullinated peptide-antibody, N (%)	307 (86.0%)	26 (81.3%)	281 (86.5%)	0.59
Methotrexate, N (%)	338 (94.7%)	28 (87.5%)	310 (95.4%)	0.14
Disease duration, years	8.21 ± 7.58	6.03 ± 5.75	8.43 ± 7.72	0.035*
Anti-TNF				
Adalimumab, N (%)	60 (16.8%)	5 (15.6%)	55 (16.9%)	1.0
Etanercept, N (%)	260 (72.8%)	23 (71.9%)	237 (72.9%)	1.0
Golimumab, N (%)	19 (5.3%)	3 (9.4%)	16 (4.92%)	0.51
Infiximab, N (%)	18 (5.0%)	1 (3.1%)	17 (5.2%)	0.92
DAS28				
Baseline	6.24 ± 0.89	6.08 ± 0.86	6.26 ± 0.90	0.28
Δ DAS28	2.48 ± 1.31	0.34 ± 0.76	2.78 ± 1.06	$< 2.2\times10^{-16}$*
CDAI				
Baseline	32.85 ± 11.83	27.67 ± 7.76	33.60 ± 12.14	8.0×10^{-3}*
Δ CDAI	21.52 ± 12.91	3.60 ± 9.87	24.1 ± 11.13	$< 3.9\times10^{-14}$*

†Each of p-values provided was obtained by either the chi-square test using categorical data or the t-test in numerical data.

*Significant (<0.05).

SD, standard deviation, BMI, Body mass index, TNF, tumor necrosis factor, DAS28, Disease Activity Scores based on 28 joint counts, CDAI, Clinical disease activity index.

Table S2 The number of variants and subjects in a quality control (QC) procedure. We note that all the 370 subjects treated with TNF-α blockers were retained and used to call copy number variation in subsequent analyses.

Table S2a Sample QC Summary

QC step	Samples removed, N	Samples retained, N
		922 (initial size)
Low call rate (< 95%)	0	922
Excessive heterozygosity	4	918
Multidimensional scale	16	902
Excessive singleton	1	901
Cryptic relatedness	1	900
Gender mismatch	0	900
Summary	22	900

Table S2b Marker QC Summary

QC step	Variants removed, N	Variants retained, N
		2,623,498 (initial size)
Duplicated SNPs	48,514	2,574,984
Low call rate (< 95%), HWE ($P_{HWE} < 1x10^{-6}$)	110,058	2,464,926
Summary	158,572	2,464,926

HWE, Hardy Weinberg equilibrium
